# Pediatric sleep apnea and depressive disorders risk: A population-based 15-year retrospective cohort study

**DOI:** 10.1371/journal.pone.0181430

**Published:** 2017-07-14

**Authors:** Chun-Hung Chang, Shaw-Ji Chen, Chieh-Yu Liu

**Affiliations:** 1 Department of Psychiatry, China Medical University Hospital, Taichung, Taiwan, R.O.C; 2 Institute of Clinical Medicine, China Medical University, Taichung, Taiwan, R.O.C; 3 Department of Nursing, Cardinal Tien Junior College of Healthcare and Management, Taipei, Taiwan, R.O.C; 4 Department of Psychiatry, Mackay Memorial Hospital Taitung Branch, Taitung, Taiwan, R.O.C; 5 Mackay Junior College of Medicine, Nursing, and Management, Taipei, Taiwan, R.O.C; 6 Institute of Medical Sciences, Tzu Chi University, Hualien, Taiwan, Taiwan, R.O.C; 7 Biostatistical Consulting Lab, Department of Midwifery and Women Health Care, National Taipei University of Nursing and Health Sciences, Taipei, Taiwan, R.O.C; 8 Department of Speech Language Pathology and Audiology, National Taipei University of Nursing and Health Sciences, Taipei, Taiwan, R.O.C; University of Texas Health Science Center at San Antonio Cancer Therapy and Research Center at Houston, UNITED STATES

## Abstract

**Background:**

Studies have shown a higher risk of depressive disorders in children with sleep apnea than in those without sleep apnea. However, the association between sleep apnea and subsequent depressive disorders in the pediatric population remains undetermined. Thus, this study investigated the risk of depressive disorders among pediatric patients with sleep apnea.

**Methods:**

In this study, the population-based National Health Insurance Research Database of Taiwan was used to identify patients who had first been diagnosed with sleep apnea between 1999 and 2013. Patients with sleep apnea who were younger than 18 years were included in the sleep apnea group. Controls (those without sleep apnea) were matched to patients with sleep apnea at a 1:10 ratio by age, sex, and index year. Patients who had baseline or inherited depressive disorders before the enrollment date were excluded. The two groups were followed up until December 31, 2013. The primary endpoint was the occurrence of one or more depressive disorders.

**Results:**

At the end of this study, 6,237 children had been enrolled, comprising 567 children with sleep apnea and 5,670 children without sleep apnea. During the mean follow-up period of 5.87 years, a total of 77 children (1.23%) developed one or more depressive disorders; 14 (2.46%) from the sleep apnea group and 63 (1.11%) from the control group. Kaplan–Meier analysis showed that children with sleep apnea had a significantly higher risk of depressive disorders (log-rank test, p = 0.002). After adjusting for covariates, the risk of subsequent depressive disorders among children with sleep apnea was still significantly higher (hazard ratio [HR] = 2.25; 95% confidence interval [CI] = 1.25–4.05; p = 0.006). Moreover, boys with sleep apnea had a significantly higher risk than those without sleep apnea (adjusted HR = 3.77; 95% CI, 1.82–7.54; p < 0.001). Furthermore, in sleep apnea group, children older than 12 years of age had more risk to depression (hazard ratio (HR) = 7.1833, 95% confidence interval (CI), 2.3734 to 21.7411; p = 0.0004).

**Conclusions:**

This study found a significantly higher subsequent risk of depressive disorders in children with sleep apnea, particularly boys and those older than 12 years of age. The study findings strongly suggest that clinicians should provide psychological evaluation and supportive care for children with sleep apnea, in addition to medical treatment.

## Introduction

Sleep apnea is characterized by repeated episodes of apnea and hypopnea during sleep, which result from the complete or partial collapse of the upper airway. The prevalence of sleep apnea in children ranges from 1% to 4% according to the varying criteria of diagnostic studies, and it is more common in boys than in girls [1]. Previous studies have demonstrated an association between sleep apnea and cardiovascular diseases, neurocognitive dysfunction, and behavioral disorders[2, 3].

As is found in adults, pediatric patients with sleep apnea may have substantial comorbid mental disorders such as depression or attention deficit hyperactivity disorder (ADHD) [4, 5]. An increase in depressive symptoms among pediatric patients with sleep apnea has been note. Studies have shown increasing incidence rates of depressive symptoms among pediatric patients with sleep apnea [6–16]. However, depression was based on scale diagnosis rather than clinical diagnosis. Depressive disorders are widespread chronic diseases that are characterized by sadness or irritability and are accompanied by several psychophysiological symptoms [17]. The long-term risk of physician-diagnosed depressive disorders among pediatric patients with sleep apnea remains unknown.

This study investigated the risk of depressive disorders among pediatric patients (aged less than 18 years) with sleep apnea by using the population-based database of the National Health Insurance Research Database (NHIRD) of Taiwan.

## Methods

### Data source

The National Health Insurance (NHI) program is a mandatory universal health insurance program launched by the Taiwanese government in 1995, and it provides comprehensive medical services to almost all residents of Taiwan. The National Health Research Institute (http://nhird.nhri.org.tw/en) is responsible for maintaining the claims data of the NHI program, which is held in the NHIRD. The NHIRD contains the health care insurance data of 98.29% of Taiwan’s population of 23 million. The National Health Research Institute validates the released database, which is representative of the entire Taiwanese population. The diagnostic and procedure codes used are based on the International Classification of Diseases, Ninth Revision, Clinical Modification (ICD-9-CM). Our study was approved by the Institutional Review Board of China Medical University Hospital (CMUH103-REC3-077).

### Study sample and controls

In this study, we identified pediatric patients who were newly diagnosed with sleep apnea (ICD-9-CM codes 780.51, 780.53, and 780.57) from 1999 to 2013 of the database, and enrolled them as the sleep apnea group. In Taiwan, a diagnosis of sleep apnea was made according to polysomnography (PSG) by board-certified pediatricians and physicians. In this study, only those having at least three consecutive corresponding diagnoses were designated as having sleep apnea for better diagnostic validity. We defined the enrollment date as the date when sleep apnea was initially diagnosed. Controls (those without sleep apnea) were selected from the NHIRD using the simple random sampling method. Controls were matched to patients in the sleep apnea group at a 1:10 ratio by age, sex, and index year. Pediatric patients who were diagnosed with a depressive disorder (ICD-9-CM codes 296.2X-296.3X, 300.4, and 311.X) before the enrollment date were excluded.

Matching for the age and year of enrollment was allowed within a tolerance range of ±1 year. For the control group, the beginning date of a follow-up was defined as the first date of visit to a clinic or hospital within the enrollment year.

### Main outcome measures

The primary endpoint of this study was the occurrence of one or more depressive disorders. Both groups were followed up until the first diagnosis of depressive disorder, death, withdrawal from the NHI program, or the study end date of December 31, 2013. In Taiwan, a diagnosis of depressive disorders was made according to ICD-9 CM code and Diagnostic and Statistical Manual of Mental Disorders (DSM)–IV [18] by board-certified psychiatrists and physicians. In this study, only those having at least three consecutive corresponding diagnoses were designated as having depressive disorders for better diagnostic validity.

### Variables

In this study, demographic characteristics—such as age, sex, and index year—were retrieved and matched between the two groups. According to previous studies, the risk factors for depressive disorders and the major comorbidities linked to pediatric sleep apnea include hypertension, diabetes mellitus, insomnia, ADHD, and asthma, which were assessed in our analyses. The Charlson comorbidity index score, which is representative of the baseline comorbidity profile, was also calculated.

### Statistical analysis

We used descriptive statistics to investigate demographic characteristics. To investigate the risk factors for depressive disorders, patients were divided into two groups—pediatric patients with sleep apnea (sleep apnea group) and pediatric patients without sleep apnea (control group). A Cox proportional hazard regression model was used to investigate the risk factors for sleep apnea. MY Structured Query Language was used for the extraction, linkage, and processing of data in the study database. All statistical analyses were performed using IBM SPSS statistical software (version 20.0 for Windows; IBM Corp., New York, NY). A two-tailed p value of <0.05 was considered statistically significant.

## Results

### Demographic characteristics

**Fig 1:**
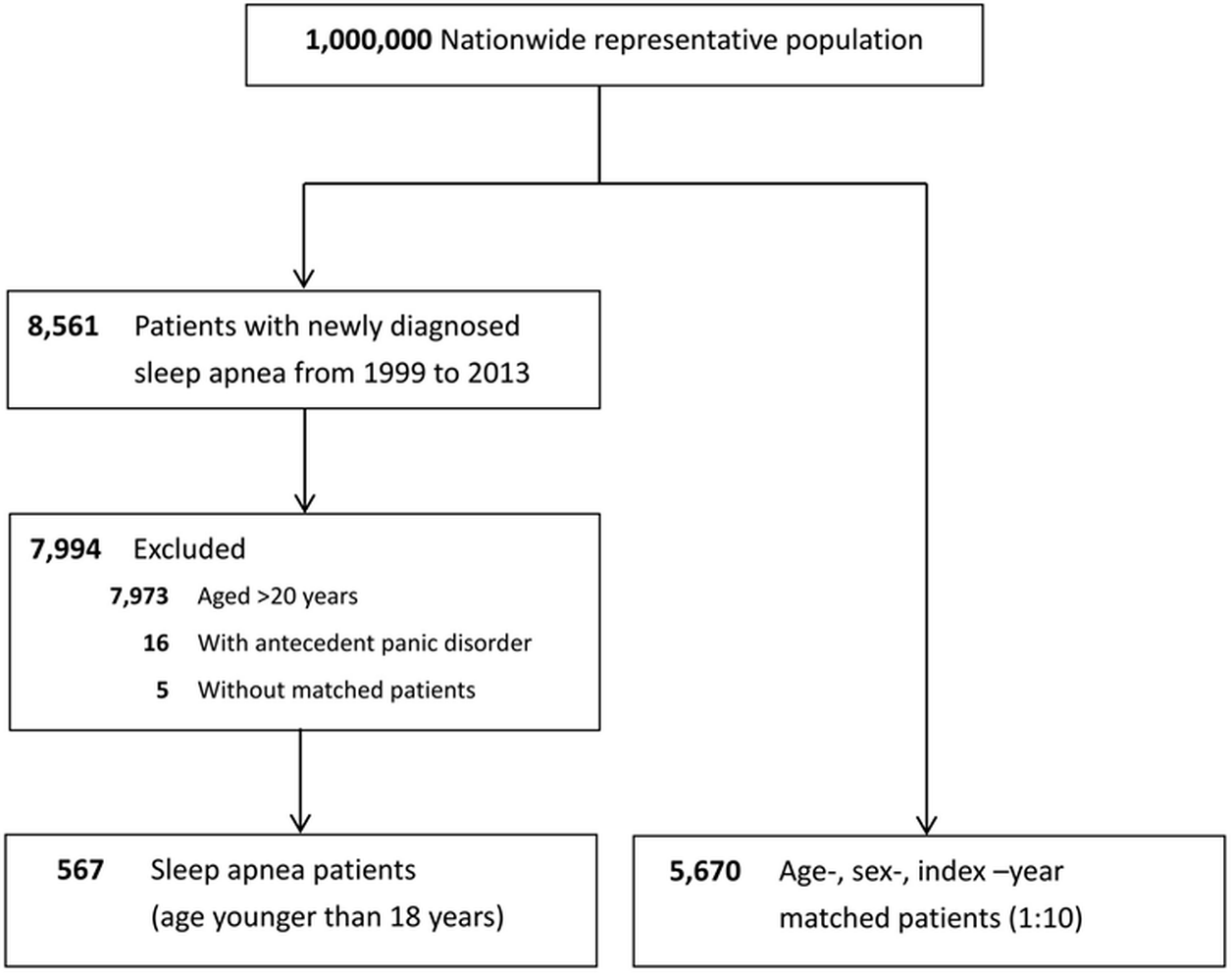
Flowchart summarizing the process of enrollment and follow-up.

A total of 567 pediatric patients with sleep apnea and 5,670 pediatric patients without sleep apnea were included in the sleep apnea and control groups, respectively. The flowchart for enrollment is shown in Fig 1. The demographic characteristics of the sleep apnea and control groups are shown in Table 1. Compared with the control group, the sleep apnea group had more comorbidities and higher Charlson comorbidity index scores.

**Table 1:** Demographic profile of study patients (n = 6,237).

		Sleep Apnea		Non-Sleep Apnea			
		(n = 567)		(n = 5,670)			
		M±SD/n(%)		M±SD/n(%)		*t*/χ^2^	p-value
Age (yrs)		9.70±4.21		9.70±4.20		0.966	1.000
Sex							
Men		381(67.19)		3810(67.2)			1.000
Women		186(32.80)		1860(32.8)			1.000
Follow up, y		5.52±3.74		5.91±3.74		0.949	0.017
Major coexisting diseases							
	Hypertension	2	(0.35)	0	(0.00)	19.326	<0.001
	Diabetes	3	(0.52)	20	(0.35)	0.088	0.766
	Insomnia	4	(0.70)	6	(0.10)	8.136	0.004
	ADHD	51	(8.99)	177	(3.12)	48.827	<0.001
	Obesity	17	(2.99)	16	(0.28)	67.180	<0.001
	Asthma	151	(26.63)	1069	(18.85)	19.326	<0.001
Charlson comorbidity score		0.021±0.166		0.015±0.137			0.421

Abbreviations: ADHD = Attention deficit hyperactivity disorder.

### Cumulative incidence of depressive disorders

**Fig 2:**
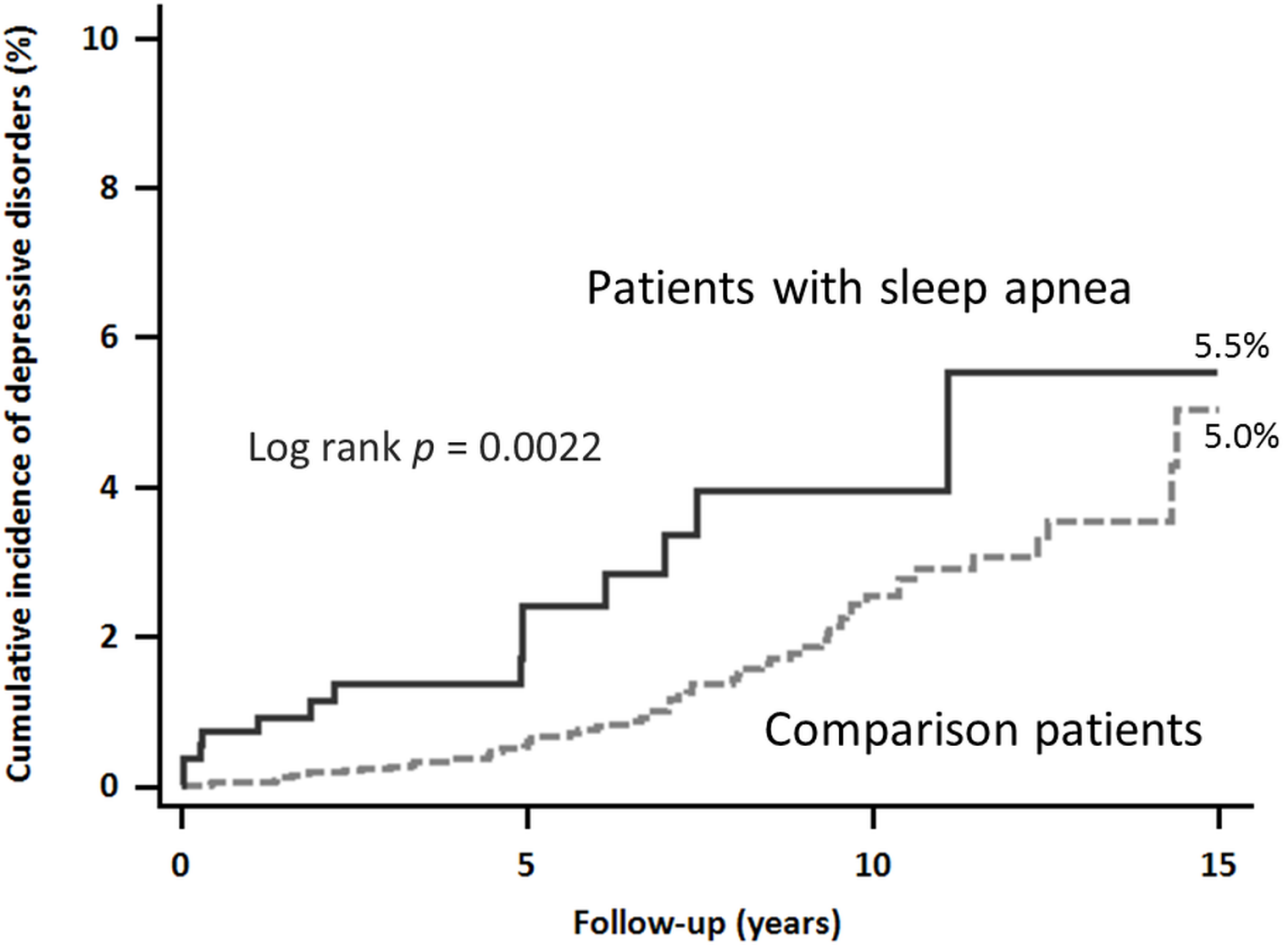
Cumulative incidence of depressive disorders in pediatric patients with sleep apnea.

**Fig 3:**
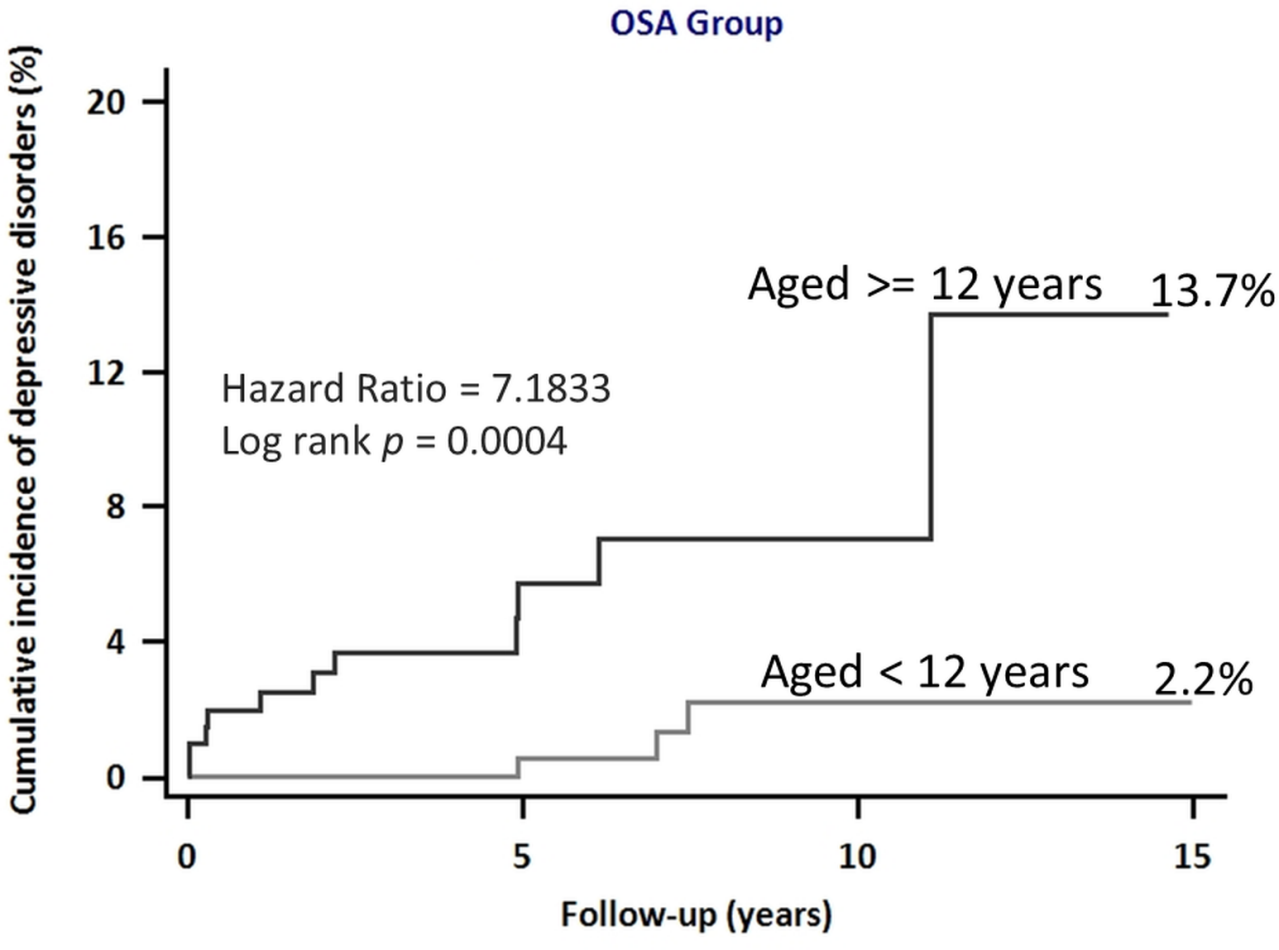
Cumulative incidence of depressive disorders after sleep apnea in two age groups.

The mean follow-up period was 5.87 years. The sleep apnea group had a higher rate of depressive disorders than the control group (14 [2.46%] vs 63 [1.11%]; p < 0.001). The sleep apnea group had a significantly higher risk of depressive disorders than the control group (log-rank test, p < 0.001, Fig 2). The incidence of depressive disorders in the sleep apnea and control groups was 181.0 and 169.0 per 1,000 person–years, respectively (p < 0.001). Comparing patients with and without depressive disorders, patients with incident depressive disorders were mostly male (57.14%) and had higher incidence of hypertension, diabetes mellitus, insomnia, ADHD, obesity, and asthma. The results of multivariate analysis indicated that sleep apnea was independently associated with incident depressive disorders (Table 2; adjusted hazard ratio [aHR] = 2.25; 95% confidence interval [CI] = 1.25–4.05; p = 0.006). In addition, boys with sleep apnea had a significantly higher risk than those without sleep apnea (aHR = 3.77; 95% CI = 1.82–7.54; p < 0.001) (Table 3). Moreover, in sleep apnea group, children older than 12 years of age had more risk to depression (hazard ratio (HR) = 7.1833, 95% confidence interval (CI), 2.3734 to 21.7411; p = 0.0004). (Fig 3)

**Table 2:** Hazard ratios of depressive disorders among study patients during 15-year follow-up period (n = 6,237).

Depressive disorders	Total (n = 6,237)	Patients with sleep apnea (n = 567)	Comparison patients (n = 5,670)	p Value
n (%)	77 (1.23)	14 (2.46)	63 (1.11)	-
Crude HR (95% CI)	-	2.40** (1.34–4.29)	1.00	0.003
Adjusted HR (95% CI) ^a^	-	2.25** (1.25–4.05)	1.00	0.006

HR, hazard ratio. HR was calculated by stratified Cox proportional hazard regressions (stratified by sex)

*p < 0.05.

**p < 0.01.

^a^ Adjustments are made for age, sex, hypertension, diabetes, insomnia, ADHD, obesity, asthma, and Charlson comorbidity score.

**Table 3:** Hazard ratios of depressive disorder by gender among the sample patients during the 15-year follow-up periods.

	Male		Female	
Depressive disorders	Patients with sleep apnea (n = 381)	Comparison patients (n = 3,810)	Patients with sleep apnea (n = 186)	Comparison patients (n = 1,860)
n (%)	11 (2.89)	33 (0.87)	3 (1.61)	30 (1.61)
Crude HR (95% CI)	3.66*** (1.85 to 7.26)	1.00	1.05 (0.32 to 3.45)	1.00
Adjusted HR (95% CI) ^a^	3.77*** (1.82 to 7.54)	1.00	1.05 (0.32 to 3.44)	1.00

HR, hazard ratio. HR was calculated by stratified Cox proportional hazard regressions (stratified by sex)

*p < 0.05

**p < 0.01

***p < 0.001

^a^ Adjustments are made for age, sex, hypertension, diabetes, insomnia, ADHD, obesity, asthma, and Charlson comorbidity score.

## Discussion

To the best of our knowledge, the present study is the first and largest cohort study to analyze the subsequent risk of depressive disorders in adolescent patients (<18 years of age) with sleep apnea. The results of this nationwide, population-representative cohort study showed that (1) the risk of depressive disorders was higher in children with sleep apnea, (2) the risk of depressive disorders was higher in boys, and (3) compared to children younger than 12 years of age, children older than 12 years of age had more risk to depression.

Our findings showed that the sleep apnea group had a higher risk of depressive disorders than the control group (14 [2.46%] vs 63 [1.11%]; p < 0.001). This result is consistent with those of previous studies. A meta-analysis of 11 studies assessed depressive symptoms in both children diagnosed with obstructive sleep apnea (OSA) (n = 894) and a comparison group (n = 1,096) and revealed a significant relationship between depressive symptoms and OSA (Hedges’ g = 0.43; 95% CI = 0.22–0.64; p = 0.00005) [6]. However, the studies in that meta-analysis addressed depressive symptoms, whereas the present study investigated physician-diagnosed depressive disorders.

The cumulative incidence between OSA group and non-OSA group was 5.5% and 5% respectively. There are some possible reasons. First, children with OSA may have more clinic visits and then result in more opportunity to be diagnosed depression by physician. This may result in the faster onset of depression in OSA group than the non-OSA group. Second, OSA treatment may reduce the depression risk. A meta-analytic study assessed 379 children across nine studies for depressive symptoms both pre- and post-surgery. Their finding shows the overall effect size (ES) change between pre- versus post-surgery was 0.41 (95% CI = 0.20–0.62; p ≤ 0.001), indicating a medium improvement occurred in depressive symptoms after adenotonsillectomy(AT). Further study may need to investigate the OSA severity and treatment effect.[6]

Several explanations have been provided for the association between sleep apnea and depressive disorders. First, sleep apnea is associated with blood oxygen desaturation during night time, which may cause daytime fatigue and depressive symptoms [19, 20]. Second, hypoxia resulting from desaturation might also lead to structural changes in the brain, which in turn lead to depressive symptoms [21]. Third, changes in hormones such as leptin may lead to depressive symptoms [22]. Leptin is involved in the regulation of food intake; therefore, leptin insensitivity may lead to obesity [23]. Obesity is a risk factor for both sleep apnea and depression[24]. Fourth, sleep apnea has been linked to low serotonin levels, which are associated with depression [21, 25].

In the present study, we found that boys with sleep apnea had a higher risk of depressive disorders than boys without sleep apnea (aHR = 3.77; 95% CI = 1.82–7.54; p < 0.001). Yilmaz et al. reported that a higher percentage of male patients had a higher effect size (beta = 0.66, p = 0.03), and their finding suggested that the relationship between depressive symptoms and sleep apnea is stronger for boys than for girls [6]. This stronger relationship may result from obesity, which is more strongly associated with depression in male patients than in female patients during adolescence[26].

The strength of our study is its nationwide, population-based cohort design. All respiratory and psychiatric disorders are recorded in the database. Thus, we could trace all cases of newly diagnosed sleep apnea and depressive disorders. Moreover, we used a large sample size to detect differences between the two groups with statistical power.

Our study had several limitations. First, the NHIRD does not provide some relevant data including body mass index, smoking history, genetic or environmental factors, which are potential confounders associated with the risk of depressive disorders. Therefore, we used a nationwide database to lower the sample bias as much as possible. Second, the patients in this study were mainly of Chinese descent. The results of this study might not be generalizable to other ethnic populations. Third, sleep apnea types, polysomnography reports, obstructive apnea hypopnea index, or adenotonsillectomy records are not kept in the database. Besides, the number of children having HTN, DM and insomnia is so small that the bias could not be excluded. Further study may need more large sample to evaluate these variables when analyzing the relationship between sleep apnea and depressive disorders.

Our study showed that pediatric patients with sleep apnea have a significantly higher risk of depressive disorders, and the risk is higher in boys and those older than 12 years of age. Clinicians should be aware that depressive disorders are possible serious comorbidities in pediatric patients with sleep apnea.

## References

[1] Ferini-Strambi L, Fantini ML, Castronovo C. Epidemiology of obstructive sleep apnea syndrome. Minerva Med. 2004;95(3):187–202. . 15289748

[2] Wallace A, Bucks RS. Memory and obstructive sleep apnea: a meta-analysis. Sleep. 2013;36(2):203–20. doi: 10.5665/sleep.2374 ; PubMed Central PMCID: PMC3543053. 23372268 10.5665/sleep.2374 PMC3543053

[3] Wang X, Ouyang Y, Wang Z, Zhao G, Liu L, Bi Y. Obstructive sleep apnea and risk of cardiovascular disease and all-cause mortality: a meta-analysis of prospective cohort studies. Int J Cardiol. 2013;169(3):207–14. doi: 10.1016/j.ijcard.2013.08.088 . 24161531 10.1016/j.ijcard.2013.08.088

[4] Lin WC, Winkelman JW. Obstructive sleep apnea and severe mental illness: evolution and consequences. Curr Psychiatry Rep. 2012;14(5):503–10. doi: 10.1007/s11920-012-0307-6 . 22872493 10.1007/s11920-012-0307-6

[5] Sedky K, Bennett DS, Carvalho KS. Attention deficit hyperactivity disorder and sleep disordered breathing in pediatric populations: a meta-analysis. Sleep Med Rev. 2014;18(4):349–56. doi: 10.1016/j.smrv.2013.12.003 . 24581717 10.1016/j.smrv.2013.12.003

[6] Yilmaz E, Sedky K, Bennett DS. The relationship between depressive symptoms and obstructive sleep apnea in pediatric populations: a meta-analysis. J Clin Sleep Med. 2013;9(11):1213–20. doi: 10.5664/jcsm.3178 ; PubMed Central PMCID: PMC3805811. 24235907 10.5664/jcsm.3178 PMC3805811

[7] Beebe DW, Ris MD, Kramer ME, Long E, Amin R. The association between sleep disordered breathing, academic grades, and cognitive and behavioral functioning among overweight subjects during middle to late childhood. Sleep. 2010;33(11):1447–56. ; PubMed Central PMCID: PMC2954694. 21102986 10.1093/sleep/33.11.1447 PMC2954694

[8] Blunden S, Lushington K, Kennedy D, Martin J, Dawson D. Behavior and neurocognitive performance in children aged 5–10 years who snore compared to controls. J Clin Exp Neuropsychol. 2000;22(5):554–68. doi: 10.1076/1380-3395(200010)22:5;1-9;FT554 . 11094391 10.1076/1380-3395(200010)22:5;1-9;FT554

[9] Bourke RS, Anderson V, Yang JS, Jackman AR, Killedar A, Nixon GM, et al Neurobehavioral function is impaired in children with all severities of sleep disordered breathing. Sleep Med. 2011;12(3):222–9. doi: 10.1016/j.sleep.2010.08.011 . 21324739 10.1016/j.sleep.2010.08.011

[10] Carotenuto M, Esposito M, Parisi L, Gallai B, Marotta R, Pascotto A, et al Depressive symptoms and childhood sleep apnea syndrome. Neuropsychiatric disease and treatment. 2012;8:369–73. doi: 10.2147/NDT.S35974 ; PubMed Central PMCID: PMC3430390. 22977304 10.2147/NDT.S35974 PMC3430390

[11] Huang YS, Guilleminault C, Li HY, Yang CM, Wu YY, Chen NH. Attention-deficit/hyperactivity disorder with obstructive sleep apnea: a treatment outcome study. Sleep Med. 2007;8(1):18–30. doi: 10.1016/j.sleep.2006.05.016 . 17157069 10.1016/j.sleep.2006.05.016

[12] Kurnatowski P, Putynski L, Lapienis M, Kowalska B. Physical and emotional disturbances in children with adenotonsillar hypertrophy. J Laryngol Otol. 2008;122(9):931–5. doi: 10.1017/S0022215107001235 . 18047762 10.1017/S0022215107001235

[13] Landau YE, Bar-Yishay O, Greenberg-Dotan S, Goldbart AD, Tarasiuk A, Tal A. Impaired behavioral and neurocognitive function in preschool children with obstructive sleep apnea. Pediatric pulmonology. 2012;47(2):180–8. doi: 10.1002/ppul.21534 . 21905262 10.1002/ppul.21534

[14] O'Brien LM, Mervis CB, Holbrook CR, Bruner JL, Klaus CJ, Rutherford J, et al Neurobehavioral implications of habitual snoring in children. Pediatrics. 2004;114(1):44–9. . 15231906 10.1542/peds.114.1.44

[15] Rosen CL, Storfer-Isser A, Taylor HG, Kirchner HL, Emancipator JL, Redline S. Increased behavioral morbidity in school-aged children with sleep-disordered breathing. Pediatrics. 2004;114(6):1640–8. doi: 10.1542/peds.2004-0103 . 15574628 10.1542/peds.2004-0103

[16] Ting H, Wong RH, Yang HJ, Lee SP, Lee SD, Wang L. Sleep-disordered breathing, behavior, and academic performance in Taiwan schoolchildren. Sleep Breath. 2011;15(1):91–8. doi: 10.1007/s11325-010-0329-4 . 20119852 10.1007/s11325-010-0329-4

[17] Belmaker RH, Agam G. Major depressive disorder. N Engl J Med. 2008;358(1):55–68. doi: 10.1056/NEJMra073096 . 18172175 10.1056/NEJMra073096

[18] Bromet E, Andrade LH, Hwang I, Sampson NA, Alonso J, de Girolamo G, et al Cross-national epidemiology of DSM-IV major depressive episode. BMC medicine. 2011;9:90 doi: 10.1186/1741-7015-9-90 ; PubMed Central PMCID: PMC3163615. 21791035 10.1186/1741-7015-9-90 PMC3163615

[19] Mitchell RB, Kelly J. Behavior, neurocognition and quality-of-life in children with sleep-disordered breathing. Int J Pediatr Otorhinolaryngol. 2006;70(3):395–406. doi: 10.1016/j.ijporl.2005.10.020 . 16321451 10.1016/j.ijporl.2005.10.020

[20] Lam RW. Sleep disturbances and depression: a challenge for antidepressants. Int Clin Psychopharmacol. 2006;21 Suppl 1:S25–9. doi: 10.1097/01.yic.0000195658.91524.61 . 16436937 10.1097/01.yic.0000195658.91524.61

[21] Schroder CM, O'Hara R. Depression and Obstructive Sleep Apnea (OSA). Ann Gen Psychiatry. 2005;4:13 doi: 10.1186/1744-859X-4-13 ; PubMed Central PMCID: PMC1181621. 15982424 10.1186/1744-859X-4-13 PMC1181621

[22] Nakra N, Bhargava S, Dzuira J, Caprio S, Bazzy-Asaad A. Sleep-disordered breathing in children with metabolic syndrome: the role of leptin and sympathetic nervous system activity and the effect of continuous positive airway pressure. Pediatrics. 2008;122(3):e634–42. doi: 10.1542/peds.2008-0154 ; PubMed Central PMCID: PMC5117807. 18762497 10.1542/peds.2008-0154 PMC5117807

[23] Reeves GM, Postolache TT, Snitker S. Childhood Obesity and Depression: Connection between these Growing Problems in Growing Children. International journal of child health and human development: IJCHD. 2008;1(2):103–14. ; PubMed Central PMCID: PMC2568994. 18941545 PMC2568994

[24] Luppino FS, de Wit LM, Bouvy PF, Stijnen T, Cuijpers P, Penninx BW, et al Overweight, obesity, and depression: a systematic review and meta-analysis of longitudinal studies. Arch Gen Psychiatry. 2010;67(3):220–9. doi: 10.1001/archgenpsychiatry.2010.2 . 20194822 10.1001/archgenpsychiatry.2010.2

[25] Yue W, Liu H, Zhang J, Zhang X, Wang X, Liu T, et al Association study of serotonin transporter gene polymorphisms with obstructive sleep apnea syndrome in Chinese Han population. Sleep. 2008;31(11):1535–41. ; PubMed Central PMCID: PMC2579982. 19014073 10.1093/sleep/31.11.1535 PMC2579982

[26] Merikangas AK, Mendola P, Pastor PN, Reuben CA, Cleary SD. The association between major depressive disorder and obesity in US adolescents: results from the 2001–2004 National Health and Nutrition Examination Survey. J Behav Med. 2012;35(2):149–54. doi: 10.1007/s10865-011-9340-x ; PubMed Central PMCID: PMC4131677. 21479835 10.1007/s10865-011-9340-x PMC4131677

